# Odonate species occupancy frequency distribution and abundance–occupancy relationship patterns in temporal and permanent water bodies in a subtropical area

**DOI:** 10.1002/ece3.6478

**Published:** 2020-07-01

**Authors:** Samuel Renner, Marina Schmidt Dalzochio, Eduardo Périco, Göran Sahlén, Jukka Suhonen

**Affiliations:** ^1^ Laboratório de Ecologia e Evolução Universidade do Vale do Taquari –UNIVATES Lajeado Brazil; ^2^ Ecology and Environmental Science RLAS Halmstad University Halmstad Sweden; ^3^ Department of Biology University of Turku Turku Finland

**Keywords:** core–satellite species patterns, damselfly, dragonfly, neotropics, SAOR patterns, SOFD patterns

## Abstract

This paper investigates species richness and species occupancy frequency distributions (SOFD) as well as patterns of abundance–occupancy relationship (SAOR) in Odonata (dragonflies and damselflies) in a subtropical area. A total of 82 species and 1983 individuals were noted from 73 permanent and temporal water bodies (lakes and ponds) in the Pampa biome in southern Brazil. Odonate species occupancy ranged from 1 to 54. There were few widely distributed generalist species and several specialist species with a restricted distribution. About 70% of the species occurred in <10% of the water bodies, yielding a surprisingly high number of rare species, often making up the majority of the communities. No difference in species richness was found between temporal and permanent water bodies. Both temporal and permanent water bodies had odonate assemblages that fitted best with the unimodal satellite SOFD pattern. It seems that unimodal satellite SOFD pattern frequently occurred in the aquatic habitats. The SAOR pattern was positive and did not differ between permanent and temporal water bodies. Our results are consistent with a niche‐based model rather than a metapopulation dynamic model.

## Graphical abstract

In this paper, we explore species richness, species occupancy frequency distributions (SOFD), and abundance–occupancy relationship (SAOR) patterns of dragonflies (Odonata) in a neotropical subtropical grassland area, especially addressing differences between permanent and temporal water bodies. We were able to show that both types of water bodies had odonate assemblages which fitted best with the unimodal satellite SOFD pattern while the SAOR pattern was positive and did not differ between permanent and temporal water bodies. Our results are consistent with a niche‐based model rather than a metapopulation dynamic model. This is to our knowledge, the first time SOFD and SAOR patterns are tested for aquatic invertebrates in a subtropical area.

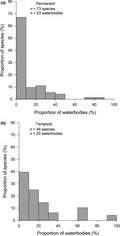

## 1 INTRODUCTION

The shape of the species occupancy frequency distribution (SOFD) and a positive species abundance–occupancy relationship (SAOR) are two widely studied patterns within community ecology (see reviews by Gaston et al., 1997, 2000; Jenkins, 2011; McGeoch & Gaston, 2002). There are two common models, which explain the SOFD (Jenkins, 2011; McGeoch & Gaston, 2002) and positive SAOR (Gaston et al., 1997; Gaston et al., 2000) patterns: (a) the metapopulation dynamic model (hereafter MPDM; Hanski, 1982; Hanski, 1999) and (b) the niche‐based model (hereafter NBM; Brown, 1984). These models are not mutually exclusive. At the ecosystem level, most species either appear at a small number of localities (satellite species; often rare) or at many localities (core species; often common and abundant), shaping the characteristic bimodal core–satellite pattern (Hanski, 1982, 1999). Through the use of a core–satellite species pattern, more information on community structure will be obtained than by analyzing only species richness. SOFDs generally have a bimodal core–satellite pattern in terrestrial habitats (Hanski, 1982, 1998, 1999; Jenkins, 2011; McGeoch & Gaston, 2002). In the case of aquatic communities, the support for this pattern is relatively weak (Verberk, van der Velde, & Esselink, 2010; Heino, 2015; but see Korkeamäki, Elo, Sahlén, Salmela, & Suhonen, 2018).

Previous studies have, however, focused on permanent aquatic communities, and it was not clear whether temporal aquatic communities would produce such pattern. Furthermore, it is unclear whether there are differences between temporal and permanent water bodies with regard to SOFD and SAOR patterns. Moreover, no studies have hitherto investigated SOFD and SOAR patterns of odonates in tropical and subtropical areas.

We used the odonate communities of water bodies of the Pampa biome in southern Brazil as a model system to test these patterns. This biome is located within the Southern Temperate Zone between latitudes 28°00' and 34°00'S and longitudes 49°30' and 58°00'W, and includes areas with both temperate and subtropical climates (Streck et al.., 2008). Recent studies have proved the existence of species‐rich odonate communities in lentic, lotic, and temporal water bodies within the Pampa biome (Renner, Périco, Dalzochio, & Sahlén, 2018; Renner, Perico, Dalzochio, & Sahlén, 2019; Renner, Périco, Ely, & Sahlén, 2017).

The aim of this study was primarily to investigate whether the odonate community species richness differed between permanent and temporal water bodies. Here, we also estimated the total species richness in both habitat types (Krebs, 1999). In addition, we determined whether the odonate community SOFD and SOAR patterns differ between permanent and temporal water bodies in the Brazilian Pampa biome. We expected that a unimodal satellite SOFD pattern would occur in both permanent and temporal water bodies, as this is frequently observed in other aquatic communities (Heino, 2015; Korkeamäki et al., 2018; Verberk et al., 2010). We also assumed that the SAOR patterns for both temporal and permanent water bodies would be positive, since this is a commonly occurring pattern (e.g. Gaston et al., 1997; 2000). In the final step, we assessed which of the two models (MPDM or NBM) best explains the observed SOFD and SAOR patterns.

## 2 MATERIAL AND METHODS

### 2.1 Data, methods, and study area

**FIGURE 1:**
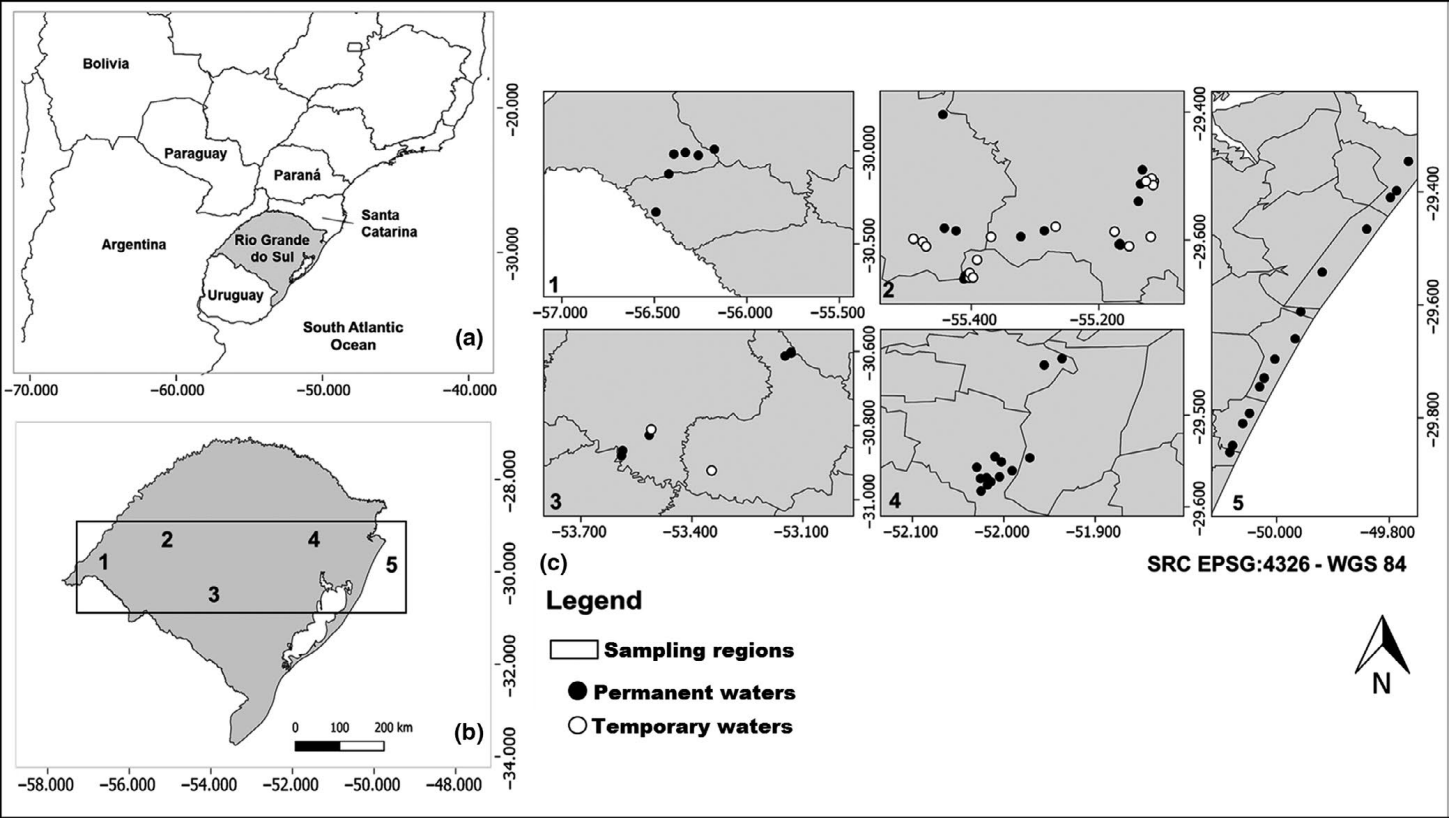
Location of Rio Grande do Sul (a), portion of the Pampa biome and the sampled regions (b), and collection sites marked with black dots (permanent) and open dots (temporary) water bodies (c)

Originally, most of the Brazilian Pampa biome (Figure 1) consisted of grassland (hence, the Pampa is treated by many authors as the “Southern Fields”), and of sparse shrub and forest vegetation (Overbeck, Müller, Pillar, & Pfadenhauer, 2009). Many areas within this biome have, however, been changed by human activities, mainly agriculture, cattle farming, and silviculture (Baldi & Paruelo, 2008; Overbeck et al., 2013). This biome is still one of the least protected in Brazil: Oliveira et al. (2017) note that only 0.8% of the Pampa is protected.

Data from 53 permanent and 20 temporal lakes and ponds (water bodies) were used (Figure 1; Renner et al., 2018, 2019). We sampled adult dragonflies from March 2011 to April 2017, visiting the localities from one (temporary waters) up to seven times during this period. We followed the method described by Renner et al., (2018), Renner et al., (2019); cf. original publications for more detailed information), collecting dragonflies on sunny days during the peak period of odonate activity (between 09:00 hr and 16:00 hr). Two persons using handheld insect nets walked along the perimeter of the sites, along the water edges and marginal zones. The average time spent at each sampling site was 45 min. This sampling method is opportunistic, and although its efficiency is constant, the probability of detecting the rarest species is reduced. Several papers discuss the problem of detecting all species at a given site (Bried, D'Amico, & Samways, 2012; Bried, Hager, et al., 2012; Hardersen, Corezzola, Gheza, Dell'Otto, & La Porta, 2017; Hedgren & Weslien, 2008; Raebel, Merckx, Riordan, Macdonald, & Thompson, 2010), highlighting the importance of detecting also rare species (Cao, Williams, & Williams, 1998). Mao and Colwell (2005) pointed out that there is only a small chance to detect the rarest species at a site, but that modern modeling approaches combined with iterative sampling seems to be a way forward (Young et al., 2019). In order to ascertain the species occupancy relationship patterns, it is crucial to show whether the number of rare species (satellite species; see below) is high or low. The impact on the results of a possible underestimate of the number of rare species due to incomplete sampling is addressed in the discussion.

Another limitation of our method is that temporary waters cannot be sampled repeatedly over a number of months (as they dry out). Although most of our permanent sites were visited repeatedly, it is therefore impossible to test for temporal variation among our samples. Renner, Sahlén, and Périco (2016) showed, for a smaller dataset within the same area, that although some of the species were seasonal, the species composition remained relatively similar throughout the year (the Sørensen index 0.73–0.83).

### 2.2 Statistical methods

Although blunt compared to more complicated hierarchical multispecies models (Iknayan, Tingley, Furnas, & Beissinger, 2014), our opportunistic data were better suited for the traditional jackknife method to estimate species richness data in temporal, permanent, and combined water bodies, and a 95% confidence interval was applied to each type of water body separately (see more details in Krebs, 1999). The jackknife species richness estimate builds on the frequency of rare species observed within the community. Here, each odonate species was recorded as present (1) or absent (0) in each water body. We also calculated the number of unique species, defined as occurring in only one water body. We used the equation by Heltshe & Forrester (1983) to calculate the estimated species richness:

$$\hat{S}=s+\left(\left(n‐1\right)/n\right)*k$$

where *Ŝ* = jackknife estimate of species richness, *s* = observed total number of species present in n water bodies, *n* = number of water bodies in total, and *k* = total number of unique species in n water bodies. For each water body we also estimated the species richness with Chao1 method in R‐package “vegan” v.2.4‐2.

We used the Moran I index to test spatial autocorrelation between the faunistic similarity and the geographical distance between water bodies. We used both Jaccard dissimilarity and Bray dissimilarity, which based on the abundance of each individual species, indexes as a distance measure of odonate species dissimilarity and community dissimilarity, respectively. The Euclidean distance (in km) was used for geographical coordinates of the water bodies. The Mantel test was calculated with R‐package “vegan” v.2.4‐2., and the statistical significance was estimated running 999 permutations. As spatial autocorrelation would nullify or affect the results of the Mantel test, we tested the spatial independence of species composition at the 73 sampling sites using a Moran I analysis. We used individual species occurrences as variables in a principal component analysis (PCA), where the first axis was used as response variable to the Moran I with coordinate variables for ten different distance classes. The global Moran's *I* analysis detected no significant spatial structure of the species composition for any distance class (minimal distance class average: 0.148 degree; Moran's *I* = 0.018; *p* = .059). Hence, we can rely on the results of the Mantel test.

Following McGeoch and Gaston (2002), we used classes of 10% occupancy, and the number or percentage of odonate species in each class, to demonstrate the variation in occupancy frequency distribution between temporal and permanent water bodies (see also Korkeamäki et al., 2018). We also tested the relationship between water body area (m^2^), length of shoreline (m), and species richness, using Pearson's correlation with a log_10_ transformation to compensate for large differences in size.

We used the same approach as Korkeamäki et al. (2018), where the multimodel inference approach was applied to regressions of ranked species occupancy curves (RSOCs as in Jenkins (2011). The three data sets (using temporal, permanent, and combined data; species in rows and water bodies in columns) were processed separately based on occupancy (presence/absence) data for individual water bodies. First, we calculated the proportion of water bodies occupied by each species (occupancy frequency) using the sum of water bodies. Second, we divided the occupancy frequency of each species by the number of water bodies, resulting in the number (relative proportion) of water bodies occupied by each species. In the following step, we arranged the species in decreasing order according to their relative occupancy values, setting *R_i_* as the rank value for species *i*, from which we plotted the relative occupancy of species (*O_i_*) as functions of *R_i_* (RSOC). Finally, we evaluated whether a unimodal satellite‐dominant, a bimodal symmetrical, a bimodal asymmetrical, or a random pattern best fitted our odonate community (cf., Jenkins, 2011). We used the IBM SPSS statistical package version 23 for all statistical calculations. As in Jenkins (2011), the Levenberg–Marquardt algorithm (with 999 iterations) was used for the nonlinear regressions, estimating the parameters (*y_0_*, *a*, *b*, and *c*) of the following four equations (by means of ordinary least squares (OLS)) to find the best fitting SOFD pattern. The equations are as follows:

1. *O_i_* = *y_0_* + a*exp(‐bRi) with initial parameters *y_0_* = 0.01, *a* = 1.0, *b* = 0.01; Unimodal satellite mode (exponential concave) pattern.
2. *O_i_* = *a*/(1 + exp (‐bRi + *c*), initial parameters *a* = 1.0, *b* = −0.1, *c* = −1.0; Bimodal symmetrical (sigmoidal symmetric) pattern.
3. *O_i_* = *a*[1–exp (‐bRi^c^)], initial parameters *a* = 1.0, *b* = −1.0, *c* = −1.0; Bimodal asymmetric (sigmoidal asymmetric) pattern.
4. *O_i_* = *a*Ri +*b*, initial parameters *a* = 0.01, *b* = 0.01; Uniform (random) pattern.

We also examined the regressions graphically for homogeneity of variance, normality of residuals, and independent error terms, as well as the tails and shoulders of the data and models (see more details in Jenkins, 2011; Korkeamäki et al., 2018).

The Akaike information criterion for small sample sizes (AICc) was used to compare the four alternative models, where the one with the smallest AICc would be best, based on the Kullback–Leibler distance (Burnham & Anderson, 2000). This approach works well to detect differences between models when values for ΔAICc (= AICc_min_ – AICc*_i_*) are higher than 7 (Anderson, Burnham, & Thompson, 2000; Burnham, Anderson, & Huyvaert, 2011; Burnham & Anderson, 2000; Jenkins, 2011).

We used a generalized linear model (GLM) to investigate the relationships between number of individuals and occupancy frequency (independent variable) in the SAOR model. The model type was negative binomial distribution with log link (type III errors) (O’Hara & Kotze, 2010). Habitat preference was divided into three categories: species observed only in (a) temporary water bodies, 9 species, (b) permanent water bodies, 34 species, and (c) both types of habitats (hereafter generalist species), 39 species. In order to test differences in occupancy frequency and number of individuals between three habitat preference categories of odonate species, we applied the generalized linear model with negative binomial distribution; log link (type III errors), using habitat preference as a factor.

## 3 RESULTS

We found 82 odonate species in the 73 water bodies (Table 1). The average number of species per water body was 9.2 (± 4.2 *SD*; range 0–18). Only one permanent lake lacked odonates altogether. Species richness did not differ between temporary (9.2 ± 3.5, *n* = 20) and permanent water bodies (9.3 ± 4.4, *n* = 53) (*t* test, *t* = 0.10, *df* = 71, *p* = .917). The estimated species richness based on the Chao1 index was slightly higher than the observed species richness, but did not differ between temporary (13.5 ± 6.0, *n* = 20) and permanent water bodies (14.0 ± 11.9, *n* = 53) (*t* test, t = 0.16, *df* = 71, *p* = .875). The Jackknife estimated species richness of the temporal water bodies was 66 (57–75) odonate species (95% confidence interval). The corresponding number for permanent water bodies was 87 (79–95) species, and in the combined data, the estimated species richness was 100 (91–109) species.

**TABLE 1:** Odonate species observed in 73 water bodies in Pampa biome in Brazil

Species	Suborder	Habitat	Temporal		Permanent		Total	
			Ind.	*n*	Ind.	*n*	Ind.	*n*
*Acanthagrion ascendens*	Zygoptera	Permanent	…	…	10	2	10	2
*Acanthagrion cuyabae*	Zygoptera	Generalist	1	1	8	3	9	4
*Acanthagrion gracile*	Zygoptera	Generalist	22	9	64	24	86	33
*Acanthagrion lancea*	Zygoptera	Generalist	6	3	67	18	73	21
*Anax concolor*	Anisoptera	Permanent	…	…	1	1	1	1
*Aphylla molossus*	Anisoptera	Temporal	2	1	…	…	2	1
*Aphylla producta*	Anisoptera	Permanent	…	…	2	2	2	2
*Aphylla theodorina*	Anisoptera	Permanent	…	…	2	2	2	2
*Argentagrion ambiguum*	Zygoptera	Permanent	…	…	10	4	10	4
*Argia albistigma*	Zygoptera	Permanent	…	…	1	1	1	1
*Argia lilacina*	Zygoptera	Generalist	14	3	48	3	62	6
*Brachymesia furcata*	Anisoptera	Permanent	…	…	8	6	8	6
*Castoraeaschna* sp.	Anisoptera	Generalist	1	1	2	2	3	3
*Dasythemis mincki*	Anisoptera	Permanent	…	…	1	1	1	1
*Diastatops intensa*	Anisoptera	Permanent	…	…	9	4	9	4
*Diastatops obscura*	Anisoptera	Permanent	…	…	2	1	2	1
*Elasmothemis* sp.	Anisoptera	Permanent	…	…	1	1	1	1
*Erythemis credula*	Anisoptera	Permanent	…	…	8	5	8	5
*Erythemis peruviana*	Anisoptera	Generalist	1	1	8	8	9	9
*Erythemis plebeja*	Anisoptera	Permanent	…	…	2	2	2	2
*Erythemis* sp.	Anisoptera	Generalist	2	2	3	3	5	5
*Erythemis vesiculosa*	Anisoptera	Generalist	6	5	3	3	9	8
*Erythodiplax lygaea*	Anisoptera	Permanent	…	…	5	2	5	2
*Erythrodiplax atroterminata*	Anisoptera	Generalist	24	12	33	18	57	30
*Erythrodiplax avittata*	Anisoptera	Permanent	…	…	4	2	4	2
*Erythrodiplax hyalina*	Anisoptera	Generalist	9	4	6	4	15	8
*Erythrodiplax media*	Anisoptera	Generalist	65	15	210	39	275	54
*Erythrodiplax melanorubra*	Anisoptera	Generalist	1	1	10	2	11	3
*Erythrodiplax nigricans*	Anisoptera	Generalist	14	7	16	11	30	18
*Erythrodiplax* sp.	Anisoptera	Generalist	20	8	34	13	54	21
*Erythrodiplax umbrata*	Anisoptera	Permanent	…	…	4	3	4	3
*Erythrodipplax paraguayensis*	Anisoptera	Generalist	27	7	99	19	126	26
*Gynothemis venipunctata*	Anisoptera	Permanent	…	…	8	2	8	2
*Hetaerina rosea*	Zygoptera	Permanent	…	…	4	2	4	2
*Homeoura chelifera*	Zygoptera	Generalist	9	2	24	13	33	15
*Idiataphe longipes*	Anisoptera	Generalist	2	2	5	3	7	5
*Ischnura capreolus*	Zygoptera	Generalist	11	4	95	20	106	24
*Ischnura fluviatilis*	Zygoptera	Generalist	36	9	294	45	330	54
*Lestes bipupillatus*	Zygoptera	Generalist	3	1	18	6	21	7
*Lestes pictus*	Zygoptera	Permanent	…	…	2	2	2	2
*Macrothemis heteronycha*	Anisoptera	Generalist	8	6	7	4	15	10
*Macrothemis lutea*	Anisoptera	Temporal	4	1	…	…	4	1
*Macrothemis marmorata*	Anisoptera	Generalist	3	3	6	2	9	5
*Miathyria marcella*	Anisoptera	Generalist	2	2	4	3	6	5
*Micrathyria hesperis*	Anisoptera	Permanent	…	…	5	3	5	3
*Micrathyria longifasciata*	Anisoptera	Temporal	3	1	…	…	3	1
*Micrathyria ocellata*	Anisoptera	Generalist	3	2	24	11	27	13
*Micrathyria* sp.	Anisoptera	Permanent	…	…	3	3	3	3
*Micrathyria spuria*	Anisoptera	Permanent	…	…	1	1	1	1
*Micrathyria tibialis*	Anisoptera	Generalist	1	1	26	11	27	12
*Minagrion waltheri*	Zygoptera	Permanent	…	…	4	1	4	1
*Mnesarete pudica*	Zygoptera	Generalist	3	1	21	2	24	3
*Negriagrion* sp.	Zygoptera	Temporal	2	1	…	…	2	1
*Nephepeltia flavifrons*	Anisoptera	Generalist	1	1	14	7	15	8
*Oligoclada laetitita*	Anisoptera	Permanent	…	…	26	8	26	8
*Orthemis aequilibris*	Anisoptera	Temporal	3	2	…	…	3	2
*Orthemis ambinigra*	Anisoptera	Generalist	7	5	1	1	8	6
*Orthemis atenuata*	Anisoptera	Temporal	1	1	…	…	1	1
*Orthemis discolor*	Anisoptera	Generalist	28	12	53	26	81	38
*Oxyagrion chapadense*	Zygoptera	Generalist	1	1	2	2	3	3
*Oxyagrion rubidum*	Zygoptera	Permanent	…	…	1	1	1	1
*Oxyagrion terminale*	Zygoptera	Generalist	9	2	5	3	14	5
*Pantala flavescens*	Anisoptera	Generalist	30	18	25	15	55	33
*Perithemis icteroptera*	Anisoptera	Permanent	…	…	58	14	58	14
*Perithemis mooma*	Anisoptera	Generalist	8	5	38	22	46	27
*Planiplax erythropyga*	Anisoptera	Permanent	…	…	4	1	4	1
*Progomphus basistictus*	Anisoptera	Temporal	3	3	…	…	3	3
*Progomphus lepidus*	Anisoptera	Permanent	…	…	10	2	10	2
*Progpmphus* sp.	Anisoptera	Permanent	…	…	1	1	1	1
*Remartinia luteipennis*	Anisoptera	Generalist	1	1	1	1	2	2
*Rhionaeschna bonariensis*	Anisoptera	Generalist	2	1	2	2	4	3
*Rhionaeschna planaltica*	Anisoptera	Generalist	1	1	4	4	5	5
*Staurophlebia reticulata*	Anisoptera	Temporal	1	1	…	…	1	1
*Tauriphila argo*	Anisoptera	Permanent	…	…	3	2	3	2
*Telebasis carmesina*	Zygoptera	Permanent	…	…	1	1	1	1
*Telebasis corallina*	Zygoptera	Generalist	2	1	47	11	49	12
*Telebasis theodori*	Zygoptera	Permanent	…	…	1	1	1	1
*Telebasis willinki*	Zygoptera	Permanent	…	…	9	4	9	4
*Tholymis citrina*	Anisoptera	Temporal	6	4	…	…	6	4
*Tramea abdominalis*	Anisoptera	Permanent	…	…	4	4	4	4
*Tramea binotata*	Anisoptera	Generalist	4	3	10	6	14	9
*Tramea cophysa*	Anisoptera	Generalist	6	5	12	9	18	14

Note

For each of the species the following information is presented: (1) Suborder [Zygoptera (damselflies), Anisoptera (dragonflies)]. (2) Habitat [generalist species were observed in both temporal water bodies and permanent lakes, temporal species were observed only in temporal water bodies, and permanent species were observed only in permanent lakes]. (3) Number of individuals (Ind.) and number of water bodies (*n*) where each species was collected in the Temporal, Permanent, and combined data (Total), respectively.

In the pooled data, the observed and estimated (Chao1) species richness neither increased with the (log_10_ transformed) water body area (*r* = 0.02, *n* = 73, *p* = .887, and *r* = 0.14, *n* = 73, *p* = .224, respectively), nor with the shore length of the water body (*r* = −0.06, *n* = 73, *p* = .632, and *r* = 0.09, *n* = 73, *p* = .454, respectively). In the permanent water bodies, the observed and Chao1 estimated species richness was also uncorrelated with water body area (*r* = 0.08, *n* = 53, *p* = .570, and *r* = 0.18, *n* = 53, *p* = .190, respectively) and shoreline length (*r* = 0.13, *n* = 53, *p* = .345, and *r* = 0.132, *n* = 53, *p* = .345, respectively). In temporal water bodies, by contrast, the species richness decreased with water body area (*r* = −0.49, *n* = 20, *p* = .029) and length of shoreline (*r* = −0.54, *n* = 20, *p* = .014). The estimated species richness (Chao1) did, however, neither correlate with water body area (*r* = −0.30, *n* = 20, *p* = .196), nor with shore length (*r* = −0.37, *n* = 20, *p* = .112).

**FIGURE 2:**
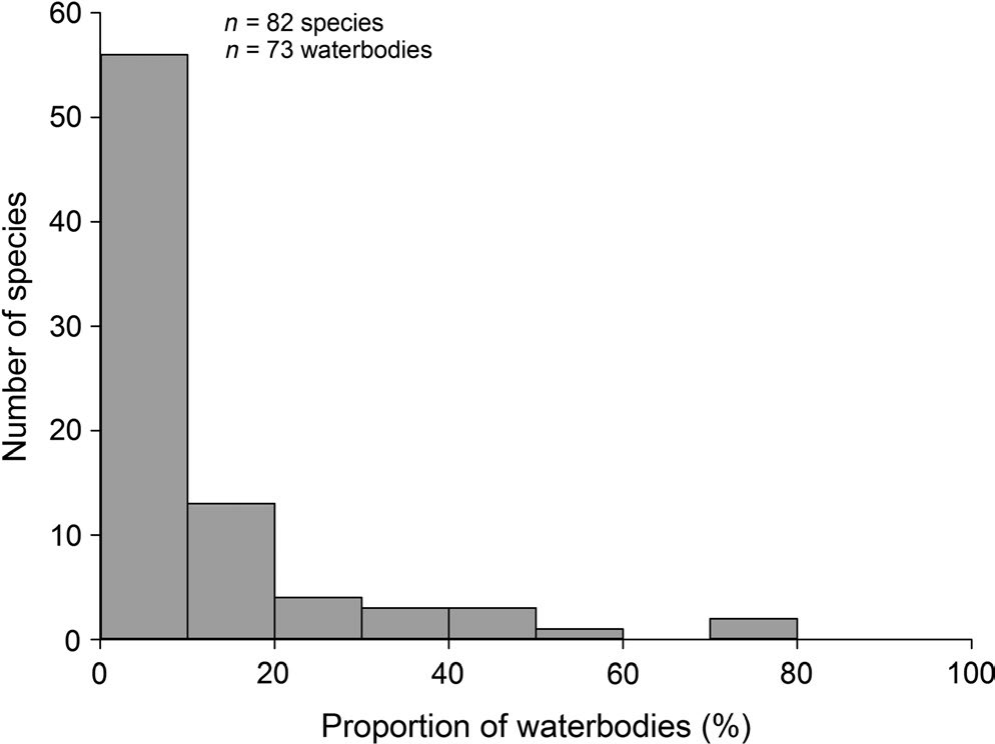
Number of odonate species (*n* = 82 species) in relation to the proportion of occupied water bodies (%) (*n* = 73 water bodies) in the Pampa biome in southern Brazil

**FIGURE 3:**
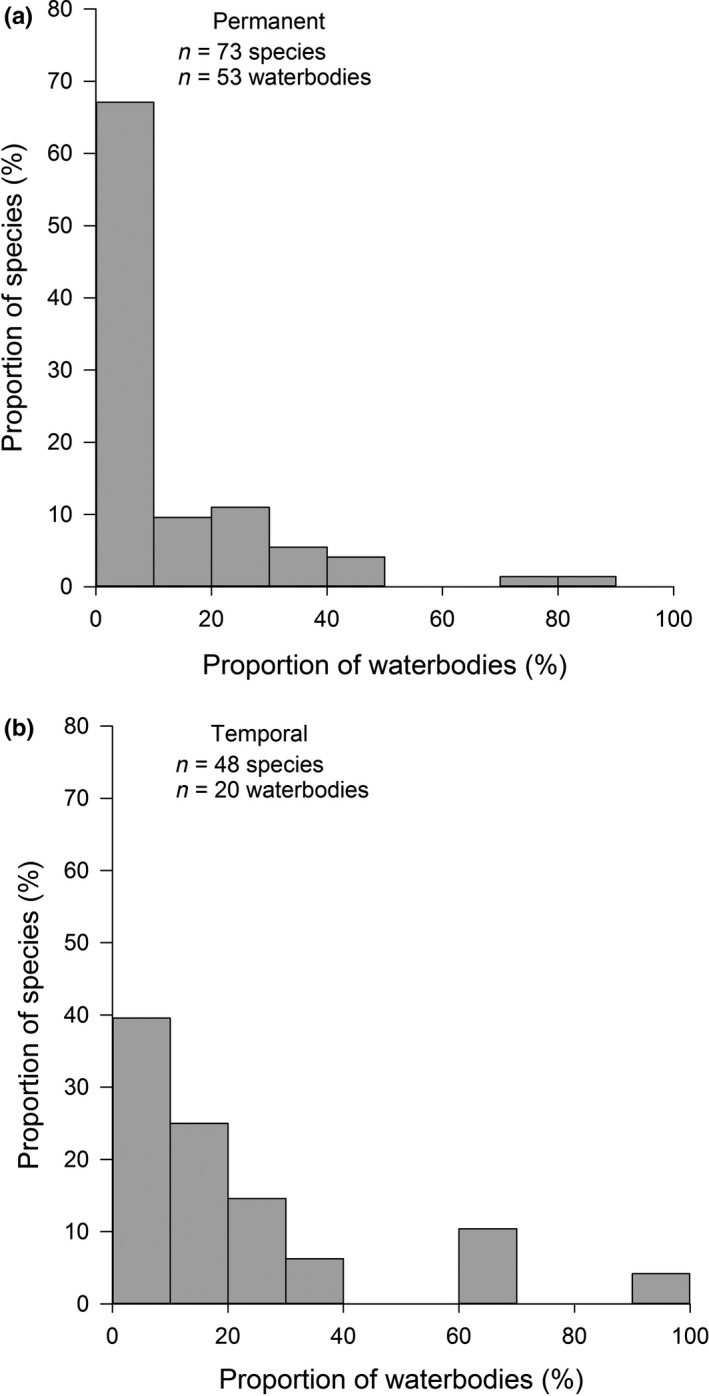
Percentage of odonate species in relation to the proportion of occupied water bodies (%) in permanent (a) and temporal (b) water bodies in the Pampa biome in Brazil

The species dissimilarity (Jaccard dissimilarity) and community dissimilarity (Bray dissimilarity) increased slightly with increasing geographical distance between water bodies (Mantel's test, *r* = 0.17, *p* = .001, and *r* = 0.16, *p* = .001, respectively). The species occurrence varied considerably, with each species occurring in 8 ± 11 (range 1 to 54) water bodies (Table 1). Most species were uncommon, with only three species occurring in at least half of the water bodies (Figure 2). The SOFD pattern was unimodal satellite in the combined data (Table 2, Figure 3). Here, we found a high number of satellite species, with two‐thirds of the species (56 out of 82) occurring in less than 10% of the water bodies. All alternative SOFD pattern models conformed less well (ΔAICc > 7; Table 2). We found no differences in the SOFD patterns between temporal and permanent water bodies (Table 2; Figure 3). In both habitat types, SOFD followed the unimodal satellite‐dominant pattern (Table 3; Figure 3). All alternative models conformed less well (ΔAICc > 7; Table 2).

**TABLE 2:** Results of odonate species occupancy frequency distributions (SOFD) in the Pampa biome in Brazil

Type of water body	Figure	Species	AICc	ΔAICc
Pooled data	2	82		
Unimodal satellite			−636.6	0
Bimodal symmetric			−585.9	50.7
Bimodal asymmetric			−474.2	162.4
Random			−376.2	260.4
Temporary	3a	48		
Unimodal satellite			−348.5	0
Bimodal symmetric			−313.1	35.4
Bimodal asymmetric			−263.0	85.5
Random			−208.7	139.8
Permanent	3b	73		
Unimodal satellite			−510.2	0
Bimodal symmetric			−477.8	32.4
Bimodal asymmetric			−438.2	72.1
Random			−328.9	181.3

Note

The four most likely SOFD patterns (random, unimodal satellite‐dominant, bimodal symmetrical, and bimodal asymmetrical) were analyzed both with combined data and separately for the temporal and permanent (lakes) water bodies. The “Figure” column joins statistical models with data figures. The “Species” column shows the number of species in each study region. AICc (Akaike information criterion for small sample sizes) as well as ΔAICc (=AICc_i_–AICc_min_) values are presented. The model with the lowest AICc is considered the best of the tested models.

**TABLE 3:** Generalized linear models for the relationships between occupancy and abundance of species in temporal, permanent, and all water bodies (pooled data) in the Pampa biome in southern Brazil

Habitat	Parameter	*SE*	Wald/*G* ^2^	*df*	*p*
Temporary					
Intercept	0.53	0.236	5.07	1	.024
Abundance	0.06	0.017	13.87	1	<.001
Model			20.77	1	<.001
Permanent					
Intercept	2.21	0.170	50.86	1	<.001
Abundance	0.02	0.005	15.31	1	<.001
Model			37.08	1	<.001
Pooled data					
Intercept	1.29	0.163	62.88	1	<.001
Abundance	0.02	0.004	21.84	1	<.001
Model			50.75	1	<.001

Note

The model type was negative binomial with log link. Estimated parameters and standard error for the intercept and predictor variable Abundance are shown. The statistical significance of the parameter was tested with Wald statistics, and the model was tested with likelihood ratio (*G* ^2^).

**FIGURE 4:**
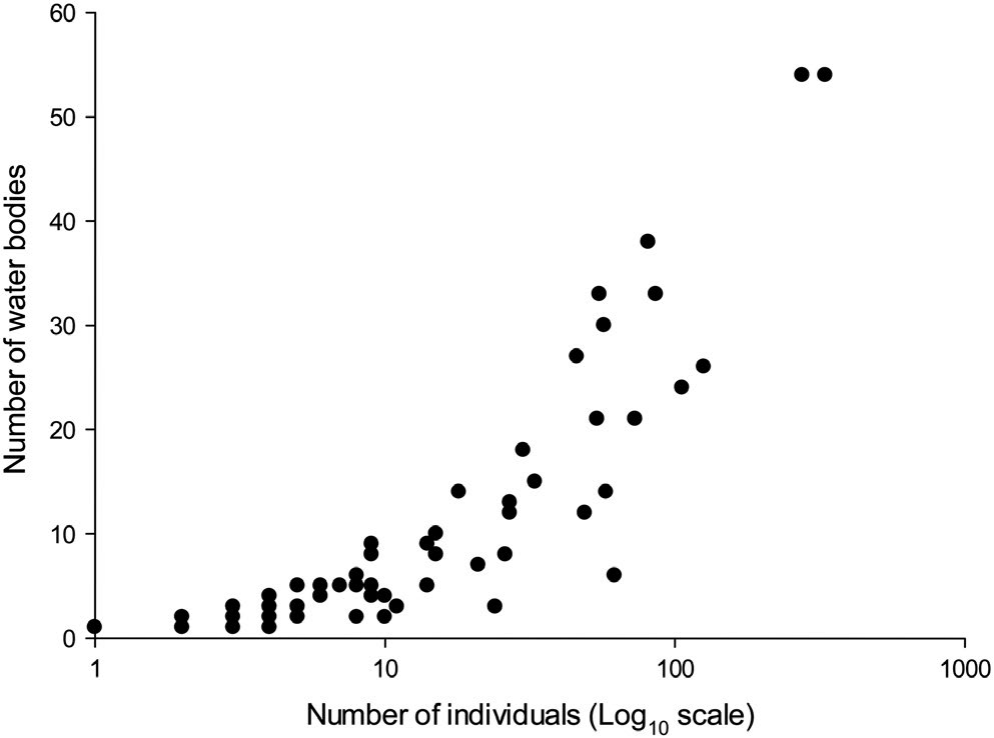
Number of observed individuals (log_10_ scale) in relation to number of water bodies occupied. Note that there are overlapping data points

All observed SAOR patterns were positive (Table 3; Figure 4). On the whole, the species represented by a high number of individuals also occurred in a larger number of both temporary and permanent water bodies, as well as in the pooled dataset (Table 3). There were differences between the three groups of species both with regard to number of occupied water bodies (GLM, *G* ^2^ = 50.95, *df* = 2, *p* < .001) and the number of individuals (GLM, *G* ^2^ = 76.72, *df* = 2, *p* < .001). Generalist species occupied a larger number of water bodies than both temporal water body species (Wald = 22.87, *df* = 1, *p* < .001; Table 4) and permanent water body species (Wald = 23.00, *df* = 1, *p* < .001; Table 3). There were no differences in species numbers between temporal and permanent water bodies (Wald = 1.37, *df* = 0.242, *p* = .242; Table 4), but generalist species were represented by a higher number of individuals than both temporal (Wald 43.53, *df* = 1, *p* < .001; Table 4) and permanent water body species (Wald = 63.44, *df* = 1, *p* < .001). In addition, permanent water body species were represented by a larger number of individuals than temporal water body species (Wald = 4.97, *df* = 1, *p* = .026).

**TABLE 4:** Estimated marginal means and 95% confidence intervals of the generalized linear model with regard to number of individuals and number of water bodies in three groups of odonate species in the Pampa biome, southern Brazil

	Mean	95% confidence intervals	
		Lower	Upper
Number of individuals			
Temporal specialist species	2.8	1.3	6.0
Permanent specialist species	6.3	4.4	9.1
Generalist species	44.7	32.5	61.4
Number of water bodies			
Temporal specialist species	1.7	0.7	3.8
Permanent specialist species	2.7	1.8	4.0
Generalist species	14.5	10.5	20.1

Note

The model type was negative binomial with log link.

## 4 DISCUSSION

### 4.1 Species richness

We found a total of 82 odonate species in the 73 studied water bodies within the Pampa biome. This is almost half (45%) of the 182 odonate species currently observed in the state of Rio Grande do Sul (Dalzochio et al., 2018; Renner et al., 2017), and c. 10% of the 854 odonate species currently recorded from Brazil (Dalzochio et al., 2018). This implies that the estimated species richness in the combined data (100 species) is realistic (i.e., <20% undetected species). Therefore, our results highlight the high odonate diversity of the Pampa biome in Brazil. Finding such a high percentage of all dragonfly species currently known from the state in our relatively small subset of Pampa biome water bodies is very interesting, especially as we recorded a large proportion of rare species (56 out of 82 species occurred in less than 10% of the water bodies). Our study thus indicates that the Pampa biome may be very species rich, a fact that the authors have also shown in a previous study (Renner et al., 2018), where rivers were also included.

Despite the species richness of the Pampa biome, we found a maximum of only 18 species per water body, and increasing water body area did not correlate with species richness. This means that our results contrast with the results of many previous studies, where larger water bodies usually harbor a larger number of odonate species (Honkanen, Sorjanen, & Mönkkönen, 2011; Korkeamäki et al., 2018; Oertli et al., 2002). Other studies in northern Europe have, however, shown that small forest lakes often harbor a higher number of species than larger lakes (Flenner & Sahlén, 2008; Koch, Wagner, & Sahlén, 2014), a pattern fitting with our Pampa biome data. In our study, almost 90% of the water bodies had a surface area of less than 1.0 ha, and only two lakes were larger than 30 ha. It seems that our area harbors a rich odonate fauna, despite the small variation in water body size.

### 4.2 Patterns in species occupancy

#### 4.2.1 Sampling artifacts

Sampling artifacts may affect SOFD and positive SAOR patterns (see discussion in Gaston et al., 2000; McGeoch & Gaston, 2002). Our results show that a unimodal satellite‐dominant SOFD pattern and a positive SAOR pattern are prevalent in the Pampa communities, both in temporary and permanent water bodies. The patterns are affected by the accuracy of the sampling (Heatherly et al., 2007; McGeoch & Gaston, 2002), but our data were gathered using a method known to detect a majority of the species present at a water body. Misidentification is also unlikely, as the authors are familiar with the Odonata of southern Brazil (cf., Bried, Hager, et al., 2012; Foster & Soluk, 2006). McGeoch and Gaston (2002) also stated that the size of the study plots influences the SOFD patterns, but in this study, we noted that the size of the water bodies did not affect the species richness. Our sample size (the number of water bodies) was large enough for both temporal and permanent water bodies (>20), and larger than the minimum suggested by McGeoch and Gaston (2002).

#### 4.2.2 Biological factors

Brown (1984) defined natural communities as having an organization where the majority of species occur either at a few sites (rare species; here: satellite species) or at numerous sites (common species; here: core species). This will result in a unimodal mode with many satellite species (cf., Heino, 2015). According to the MPDM (Hanski, 1982), the alternative bimodal core–satellite species pattern (Hanski, 1982, 1999), bimodality should result from random colonization and extinction events among the species in the local communities. Species would either be highly susceptible to extinction (the rare satellite species) or occurring relatively permanently (the abundant core species). We noted that most of the Pampa species were recorded in only a small fraction (<10%) of the water bodies and that only a few species were found in more than half of the water bodies. The unimodal satellite‐dominant SOFD pattern describes this situation well (Figure 2). The general theory of species community structure, suggested by e.g. Brown (1981) and discussed by Lennon, Koleff, Greenwood, and Gaston (2004), coincides well with the pattern observed by us: our water bodies harbor merely a small number of common species and numerous rare ones.

This SOFD pattern may be dependent on both biotic and abiotic factors, as suggested by McGeoch and Gaston (2002) and Jenkins (2011)—namely habitat disturbance, niche breadth, and dispersal ability of the species (Jokimäki, Suhonen, & Kaisanlahti‐Jokimäki, 2016; Korkeamäki et al., 2018). However, the niche breadth hypothesis (Brown, 1984), predicting a right‐skewed unimodal SOFD pattern, fits well with our results and is in accordance with previously published results for aquatic habits (Heino, 2015; Korkeamäki et al., 2018; Verberk et al., 2010). This hypothesis predicts that generalist species with broader niches often have a wider distribution area, whereas more specialized species are restricted by their smaller niches. Specialist species which occupied only temporal water bodies or permanent lakes were less common in the region. In literature, a number of authors have likewise demonstrated that species with a small distribution (rare species) are more likely to undergo local extinctions (Hanski, 1998; Korkeamäki & Suhonen, 2002; Suhonen, Korkeamäki, Salmela, & Kuitunen, 2014). This elevated local extinction risk is probably due to higher environmental vulnerability linked to smaller population size when compared to common/generalist species inhabiting the same environment (Korkeamäki & Suhonen 2002; Suhonen et al., 2010; Suhonen et al., 2014). Our results also support the hypothesis of dispersal ability, which predicts a unimodal SOFD pattern dominated by satellite species (Collins & Glenn, 1997). Although dispersal ability is insufficiently investigated in Odonata species, at least some well‐studied species have been shown, directly or indirectly, to have a very good dispersal ability (Andersen, Nilsson, & Sahlén, 2016; Suhling, Martens, & Suhling, 2017; Troast, Suhling, Jinguji, Sahlén, & Ware, 2016), sometimes being able to fly hundreds of kilometers. A few species are weak flyers (e.g., Rouquette & Thompson, 2005), but only a small number of species have been studied in detail. We may postulate that a good dispersal ability is an ecological requirement for species searching for a scarce or ephemeral reproduction habitat. An alternative explanation is that in a species‐rich area, such as the Pampa biome in Brazil, only a small part of the species pool occurs in the same local assemblage. Such a distribution may also explain our observed unimodal satellite‐dominant SOFD pattern. A previous study has, indeed, shown that the original species assemblies in the Pampa area were very probably species poor but very diverse between sites (Renner et al., 2018).

In our area, we showed that species occurring at only a small number of water bodies also had small population sizes, as measured by the number of individuals observed at the water bodies. Our data thus indicated a positive SAOR pattern, which did not differ between temporal and permanent water bodies. The few common species might be widely distributed due to a low local extinction ratio and a high colonization ratio (Hanski, 1998; Hanski & Gyllenberg, 1997).

Although both the MPDM (e.g. Hanski, 1982) and the NBM (e.g. Brown, 1984) predict a positive SAOR, our results rather tend to support the NBM. According to this model, generalist species with wider niches and a high tolerance of environmental variation are widely distributed and locally abundant. On the other hand, specialized species with narrow niches and a greater sensitivity to environmental variation occur more locally and have a limited regional distribution (Brown, 1984). According to our data, generalist species occupied a larger proportion of the water bodies when the number of individuals was accounted for, which supports NBM.

## 5 LIMITATIONS

Performing our study in the undersurveyed Brazilian Pampa provided interesting new information, but also made certain constraints apparent. First, there is the issue of the sampling efficiency, where we know we have not been able to catch all species occurring in the region. An opportunistic sampling design was employed, as it was frequently impossible to visit temporary waters more than once. This single visit type of sampling is known to limit biodiversity information in aquatic environments (Bried & Hinchliffe, 2019), but for a first survey of an area we deemed it to work sufficiently well. Calculating the SOFD patterns adding a larger number of rare species to the equation (assuming all species not detected by us to be rare) would give the same results: Adding more satellite species to the equations would still render a unimodal satellite‐dominant pattern. In addition, the fact that we were unable to conduct sampling at all seasons might affect our results, especially since temporal variation (and primary production) is suggested to be the best predictors of spatial variation (suborder Zygoptera in streams in the Amazon; Brasil et al., 2019). However, our test for autocorrelation shows that our localities are independent when it comes to species composition, suggesting an independent dispersal of the species we were able to survey. This was also noted by Bonada, Doledec, and Statzner (2012) for rivers in the Mediterranean basin, where Odonata assemblages, unlike assemblage of other taxonomic groups, were not autocorrelated. Bonada et al. (2012) highlighted a pattern where groups with high dispersal capacity form assemblages displaying low autocorrelation. This fits well with the lentic Odonata of southern Brazil.

## 6 CONCLUSIONS

To conclude, our results demonstrate a general SOFD and a positive SAOR pattern of numerous rare and few common species in the investigated Pampa biome communities. Our findings support the NBM model (Brown, 1984), because odonate assemblages are arranged in a unimodal mode, having a positive SAOR and a high number of satellite species. To achieve a better understanding of the geographical variation in SOFD for aquatic life, more experimental and theoretical research is required. The patterns regulating the range and distribution of species—and how these affect the occupancy frequency of species in lotic, lentic, and temporal water bodies as well as in different biomes—need to be investigated further. We suggest that several similar (SOFD/SAOR) studies be conducted in subtropical and tropical areas, aiming at a better understanding of occurrence patterns. This knowledge is crucial to the development of conservation measures in highly diverse environments such as these. We also recommend that further studies of species assembly patterns in this region take at least some of the limiting factors mentioned above into account.

## CONFLICT OF INTEREST

None declared.

## AUTHOR CONTRIBUTION

**Samuel Renner:** Conceptualization (equal); Data curation (equal); Formal analysis (equal); Funding acquisition (equal); Investigation (equal); Methodology (equal); Project administration (equal). **Marina Schmidt Dalzochio:** Conceptualization (equal); Data curation (equal); Formal analysis (equal). **Eduardo Périco:** Conceptualization (equal); Data curation (equal); Formal analysis (equal); Funding acquisition (equal); Investigation (equal); Methodology (equal); Project administration (equal). **Göran Sahlén:** Conceptualization (equal); Data curation (equal); Formal analysis (equal); Funding acquisition (equal); Investigation (equal); Methodology (equal); Project administration (equal). **Jukka Suhonen:** Conceptualization (equal); Data curation (equal); Formal analysis (equal); Methodology (equal).

## Data Availability

The original data are filed in the public repository Dryad under the following DOI accession number: https://doi.org/10.5061/dryad.d51c5b008.

## References

[ece36478-bib-0001] Andersen, E. , Nilsson, B. , & Sahlén, G. (2016). Survival possibilities of the dragonfly *Aeshna viridis* (Insecta, Odonata) in southern Sweden predicted from dispersal possibilities. Journal of Insect Conservation, 20, 179–188. 10.1007/s10841-016-9850-5

[ece36478-bib-0002] Anderson, D. , Burnham, K. , & Thompson, W. (2000). Null hypothesis testing: Problems, prevalence, and an alternative. Journal of Wildlife Management, 64, 912–923. 10.2307/3803199

[ece36478-bib-0003] Baldi, G. , & Paruelo, J. M. (2008). Land use and land cover dynamics in South American temperate grasslands. Ecology and Society, 13, 6 10.5751/ES-02481-130206

[ece36478-bib-0004] Bonada, N. , Doledec, S. , & Statzner, B. (2012). Spatial autocorrelation patterns of stream invertebrates: Exogenous and endogenous factors. Journal of Biogeography, 39, 56–68. 10.1111/j.1365-2699.2011.02562.x

[ece36478-bib-0005] Brasil, L. S. , Silverio, D. V. , Cabette, H. S. R. , Batista, J. D. , Vieira, T. B. , Dias‐Silva, K. , … Juen, L. (2019). Net primary productivity and seasonality of temperature and precipitation are predictors of the species richness of the Damselflies in the Amazon. Basic and Applied Ecology, 35, 45–53. 10.1016/j.baae.2019.01.001

[ece36478-bib-0006] Bried, J. T. , D'Amico, F. , & Samways, M. J. (2012). A critique of the dragonfly delusion hypothesis: Why sampling exuviae does not avoid bias. Insect Conservation and Diversity, 5, 398–402. 10.1111/j.1752-4598.2011.00171.x

[ece36478-bib-0007] Bried, J. T. , Hager, B. J. , Hunt, P. D. , Fox, J. N. , Jensen, H. J. , & Vowels, K. M. (2012). Bias of reduced‐effort community surveys for adult Odonata of lentic waters. Insect Conservation and Diversity, 5, 213–222. 10.1111/j.1752-4598.2011.00156.x

[ece36478-bib-0008] Bried, J. T. , & Hinchliffe, R. P. (2019). Improving taxonomic resolution in large‐scale freshwater biodiversity monitoring: An example using wetlands and Odonata. Insect Conservation and Diversity, 12, 9–17. 10.1111/icad.12323

[ece36478-bib-0009] Brown, J. (1981). Two decades of homage to Santa Rosalia: Toward a general theory of diversity. American Zoologist, 21, 877–888. 10.1093/icb/21.4.877

[ece36478-bib-0010] Brown, J. (1984). On the relationship between abundance and distribution of species. American Naturalist, 124, 255–279. 10.1086/284267

[ece36478-bib-0011] Burnham, K. P. , Anderson, D. R. , & Huyvaert, K. P. (2011). AIC model selection and multimodel inference in behavioural ecology: Some background, observation, and comparisons. Behavioral Ecology and Sociobiology, 65, 23–35.

[ece36478-bib-0012] Burnham, P. , & Anderson, D. R. (2000). Model selection and multimodel inference. A practical information‐theoretic approach. New York, NY: Springer, 521 p.

[ece36478-bib-0013] Cao, Y. , Williams, D. D. , & Williams, N. E. (1998). How important are rare species in aquatic community ecology and bioassessment? Limnology and Oceanography, 43, 1403–1409. 10.4319/lo.1998.43.7.1403

[ece36478-bib-0014] Collins, S. , & Glenn, S. (1997). Effects of organismal and distance scaling on analysis of species distribution and abundance. Ecological Applications, 7, 543–551. 10.1890/1051-0761(1997)007[0543:EOOADS]2.0.CO;2

[ece36478-bib-0015] Dalzochio, M. S. , Renner, S. , Sganzerla, C. , Prass, G. , Ely, G. J. , Salvi, L. C. , … Perico, E. (2018). Checklist of Odonata (Insecta) in the state of Rio Grande do Sul, Brazil with seven new records. Biota Neotropica, 18, e20180551 10.1590/1676-0611-bn-2018-0551

[ece36478-bib-0016] Flenner, I. , & Sahlén, G. (2008). Dragonfly community re‐organisation in boreal forest lakes: Rapid species turnover driven by climate change? Insect Conservation and Diversity, 1, 169–179. 10.1111/j.1752-4598.2008.00020.x

[ece36478-bib-0017] Foster, S. E. , & Soluk, D. A. (2006). Protecting more than the wetland: The importance of biased sex ratios and habitat segregation for conservation of the Hine's emerald dragonfly, *Somatochlora hineana* Williamson. Biological Conservation, 127, 158–166. 10.1016/j.biocon.2005.08.006

[ece36478-bib-0018] Gaston, K. , Blackburn, T. , Greenwood, J. , Gregory, R. , Quinn, R. , & Lawton, J. (2000). Abundance‐occupancy relationships. Journal of Applied Ecology, 37, 39–59. 10.1046/j.1365-2664.2000.00485.x

[ece36478-bib-0019] Gaston, K. J. , Blackburn, T. M. , & Lawton, J. H. (1997). Interspecific abundance‐range size relationships: An appraisal of mechanisms. The Journal of Animal Ecology, 66(4), 579–601. 10.2307/5951

[ece36478-bib-0020] Hanski, I. (1982). Dynamics of regional distribution – the core and satellite species hypothesis. Oikos, 38, 210–221. 10.2307/3544021

[ece36478-bib-0021] Hanski, I. (1998). Metapopulation dynamics. Nature, 396, 41–49. 10.1038/23876

[ece36478-bib-0022] Hanski, I. (1999). Metapopulation ecology. Oxford: Oxford University Press, 313 p.

[ece36478-bib-0023] Hanski, I. , & Gyllenberg, M. (1997). Uniting two general patterns in the distribution of species. Science, 275, 397–400. 10.1126/science.275.5298.397 8994039

[ece36478-bib-0024] Hardersen, S. , Corezzola, S. , Gheza, G. , Dell'Otto, A. , & La Porta, G. (2017). Sampling and comparing odonate assemblages by means of exuviae: Statistical and methodological aspects. Journal of Insect Conservation, 21, 207–218. 10.1007/s10841-017-9969-z

[ece36478-bib-0025] Heatherly, T. , Whiles, M. R. , Gibson, D. J. , Collins, S. L. , Huryn, A. D. , Jackson, J. K. , & Palmer, M. A. (2007). Stream insect occupancy‐frequency patterns and metapopulation structure. Oecologia, 151, 313–321. 10.1007/s00442-006-0596-8 17091283

[ece36478-bib-0026] Hedgren, O. , & Weslien, J. (2008). Detecting Rare Species with Random or Subjective Sampling: A Case Study of Red Listed Saproxylic Beetles in Boreal Sweden. Conservation Biology, 22, 212–215. 10.1111/j.1523-1739.2007.00848.x 18273954

[ece36478-bib-0027] Heino, J. (2015). Deconstructing occupancy frequency distributions in stream insects: Effects of body size and niche characteristics in different geographical regions. Ecological Entomology, 40, 491–499. 10.1111/een.12214

[ece36478-bib-0028] Heltshe, J. F. , & Forrester, N. E. (1983). Estimating Species Richness Using the Jackknife Procedure. Biometrics, 39, 1–11. 10.2307/2530802 6871338

[ece36478-bib-0029] Honkanen, M. , Sorjanen, A. , & Mönkkönen, M. (2011). Deconstructing responses of dragonfly species richness to area, nutrients, water plant diversity and forestry. Oecologia, 166, 457–467. 10.1007/s00442-010-1846-3 21113624

[ece36478-bib-0030] Iknayan, K. J. , Tingley, M. W. , Furnas, B. J. , & Beissinger, S. R. (2014). Detecting diversity: emerging methods to estimate species diversity. Trends in Ecology & Evolution, 29, 97–106. 10.1016/j.tree.2013.10.012 24315534

[ece36478-bib-0031] Jenkins, D. G. (2011). Ranked species occupancy curves reveal common patterns among diverse metacommunities. Global Ecology and Biogeography, 20, 486–497. 10.1111/j.1466-8238.2010.00617.x

[ece36478-bib-0032] Jokimäki, J. , Suhonen, J. , & Kaisanlahti‐Jokimäki, M. (2016). Urbanization and species occupancy frequency distribution pattern in core zone areas of European towns. European Journal of Ecology, 2, 23–43. 10.1515/eje-2016-0014

[ece36478-bib-0033] Koch, K. , Wagner, C. , & Sahlén, G. (2014). Farmland versus forest: Comparing changes in Odonata species composition in western and eastern Sweden. Insect Conservation and Diversity, 7, 22–31. 10.1111/icad.12034

[ece36478-bib-0034] Korkeamäki, E. , Elo, M. , Sahlén, G. , Salmela, J. , & Suhonen, J. (2018). Regional variations in occupancy frequency distribution patterns between odonate assemblages in Fennoscandia. Ecosphere, 9, e02192 10.1002/ecs2.2192

[ece36478-bib-0035] Korkeamäki, E. , & Suhonen, J. (2002). Distribution and habitat specialization of species affect local extinction in dragonfly Odonata populations. Ecography, 25, 459–465. 10.1034/j.1600-0587.2002.250408.x

[ece36478-bib-0036] Krebs, C. J. (1999). Ecological methodology (2nd edn.). Menlo Park, CA: Benjamin/Cummings, 620 p.

[ece36478-bib-0037] Lennon, J. , Koleff, P. , Greenwood, J. , & Gaston, K. (2004). Contribution of rarity and commonness to patterns of species richness. Ecology Letters, 7, 81–87. 10.1046/j.1461-0248.2004.00548.x

[ece36478-bib-0038] Mao, C. X. , & Colwell, R. K. (2005). Estimation of species richness: Mixture models, the role of rare species, and inferential challenges. Ecology, 86, 1143–1153. 10.1890/04-1078

[ece36478-bib-0039] McGeoch, M. , & Gaston, K. (2002). Occupancy frequency distributions: Patterns, artefacts and mechanisms. Biological Reviews, 77, 311–331. 10.1017/S1464793101005887 12227519

[ece36478-bib-0040] O’Hara, R. B. , & Kotze, D. J. (2010). Do not log‐transform count data. Methods in Ecology and Evolution, 1, 118–122. 10.1111/j.2041-210X.2010.00021.x

[ece36478-bib-0041] Oertli, B. , Auderset Joye, D. , Castella, E. , Juge, R. , Cambin, D. , & Lachavanne, J. (2002). Does size matter? The relationship between pond area and biodiversity. Biological Conservation, 104, 59–70. 10.1016/S0006-3207(01)00154-9

[ece36478-bib-0042] Oliveira, U. , Soares‐Filho, B. S. , Paglia, A. P. , Brescovit, A. D. , Carvalho, C. J. B. , Silva, D. P. , … Santos, A. J. (2017). Biodiversity conservation gaps in the Brazilian protected areas. Scientific Reports, 7, 9141 10.1038/s41598-017-08707-2 28831073 PMC5567310

[ece36478-bib-0043] Overbeck, G. E. , Hermann, J. M. , Andrade, B. O. , Boldrini, I. I. , Kiehl, K. , Kirmer, A. , … Pillar, V. D. (2013). Restoration ecology in Brazil – time to step out of the forest. Brazilian Journal of Nature Conservation, 11, 92–95.

[ece36478-bib-0044] Overbeck, G. E. , Müller, S. C. , Pillar, V. D. , & Pfadenhauer, J. (2009). Floristic composition, environmental variation and species distribution patterns in burned grassland in southern Brazil. Brazilian Journal of Biology, 66, 1073–1090. 10.1590/S1519-69842006000600015 17299944

[ece36478-bib-0045] Raebel, E. M. , Merckx, T. , Riordan, P. , Macdonald, D. W. , & Thompson, D. J. (2010). The dragonfly delusion: Why it is essential to sample exuviae to avoid biased surveys. Journal of Insect Conservation, 14, 523–533. 10.1007/s10841-010-9281-7

[ece36478-bib-0046] Renner, S. , Périco, E. , Dalzochio, M. S. , & Sahlén, G. (2018). Water body type and land cover shape the dragonfly communities (Odonata) in the Pampa biome, Rio Grande do Sul, Brazil. Journal of Insect Conservation, 22, 113–125. 10.1007/s10841-017-0042-8

[ece36478-bib-0047] Renner, S. , Perico, E. , Dalzochio, M. S. , & Sahlén, G. (2019). Ecoregions within the Brazilian Pampa biome reflected in Odonata species assemblies. Austral Ecology, 44, 461–472. 10.1111/aec.12680

[ece36478-bib-0048] Renner, S. , Périco, E. , Ely, G. J. , & Sahlén, G. (2017). Preliminary dragonfly (Odonata) species list from the Pampa biome in Rio Grande do Sul, Brazil, with ecological notes for 19 new records for the State. Biota Neotropica, 17(4), e20170374 10.1590/1676-0611-bn-2017-0374

[ece36478-bib-0049] Renner, S. , Sahlén, G. , & Périco, E. (2016). Testing dragonflies as species richness indicators in a fragmented subtropical Atlantic Forest environment. Neotropical Entomology, 45, 231–239. 10.1007/s13744-015-0355-9 26686194

[ece36478-bib-0050] Rouquette, J. , & Thompson, D. (2005). Habitat associations of the endangered damselfly, *Coenagrion mercuriale*, in a water meadow ditch system in southern England. Biological Conservation, 123, 225–235. 10.1016/j.biocon.2004.11.011

[ece36478-bib-0051] Streck, E. V. , Kampf, N. , Dalmolin, R. S. D. , Klamt, E. , do Nascimento, P. C. , Schneider, P. , … Pinto, L. F. S. (2008). Solos do Rio Grande do Sul (2nd edn.). Porto Alegre: EMATER/RS‐ASCAR, 222 p.

[ece36478-bib-0052] Suhling, F. , Martens, A. , & Suhling, I. (2017). Long‐distance dispersal in Odonata: Examples from arid Namibia. Austral Ecology, 42, 544–552. 10.1111/aec.12472

[ece36478-bib-0053] Suhonen, J. , Hilli‐Lukkarinen, M. , Korkeamäki, E. , Kuitunen, M. , Kullas, J. , Penttinen, J. , & Salmela, J. (2010). Local extinction of dragonfly and damselfly populations in low‐ and high‐quality habitat patches. Conservation Biology, 24, 1148–1153. 10.1111/j.1523-1739.2010.01504.x 20412087

[ece36478-bib-0054] Suhonen, J. , Korkeamäki, E. , Salmela, J. , & Kuitunen, M. (2014). Risk of local extinction of Odonata freshwater habitat generalists and specialists. Conservation Biology, 28, 783–789. 10.1111/cobi.12231 24405332

[ece36478-bib-0055] Troast, D. , Suhling, F. , Jinguji, H. , Sahlén, G. , & Ware, J. (2016). A global population genetic study of *Pantala flavescens* . PLoS One, 11, e0148949 10.1371/journal.pone.0148949 26934181 PMC4775058

[ece36478-bib-0056] Verberk, W. C. E. P. , van der Velde, G. , & Esselink, H. (2010). Explaining abundance‐occupancy relationships in specialists and generalists: A case study on aquatic macroinvertebrates in standing waters. Journal of Animal Ecology, 79, 589–601. 10.1111/j.1365-2656.2010.01660.x 20202007

[ece36478-bib-0057] Young, N. E. , Fairchild, M. , Belcher, T. , Evangelista, P. , Verdone, C. , & Stohlgren, T. J. (2019). Finding the needle in the haystack: Iterative sampling and modeling for rare taxa. Journal of Insect Conservation, 23, 589–595. 10.1007/s10841-019-00151-z

